# Review of *Bloodletting and Miraculous Cures *by Vincent Lam

**DOI:** 10.1186/1747-5341-2-14

**Published:** 2007-07-15

**Authors:** Stephanie A Nixon, Joel Baetz

**Affiliations:** 1 University of KwaZulu-Natal and University of Toronto, Health Economics and HIV/AIDS Research Division (HEARD), University of KwaZulu-Natal, Westville Campus, J Block, Level 4, University Road, Westville, Durban, South Africa; 2 York University, Department of English, 208 Stong College, 4700 Keele Street, Toronto, Ontario, M3J 1P3, Canada

## 

Few first books are fortunate enough to receive both high praise and big awards, but Vincent Lam's *Bloodletting and Miraculous Cures *deserves the attention. Celebrated by critics and awarded one of Canada's top literary prizes, *Bloodletting *is a masterful, smart and engaging debut collection of short stories. Part-time writer and full-time emergency-room physician in Toronto, Vincent Lam paints a three-dimensional portrait of physicians grappling with inner struggles, ethical dilemmas and hospital-room obscurities. The collection follows four Toronto doctors – Ming, Sri, Fitzgerald, and Chen – from hopeful undergrads to medical trainees to seasoned physicians. Through their experiences, Lam examines the myths and truths of today's health care world.

The collection's four main characters are, in the end, practicing physicians, and "practicing" is the key word here. The doctors in *Bloodletting *practice medicine; they do not perfect it. They guess at the effects of their actions. They try to interpret symptoms, reach infallible conclusions, maintain their authority, and care for their patients. But they make mistakes. They argue. Sometimes they hate their work. It is that recognition of the difficulties of providing health care that makes this collection enjoyable and important. *Bloodletting *succeeds because it challenges the myth that doctors are omnipotent and medicine is objective. Instead, this book offers a complex rendition of hospital life, one that is simultaneously valuable for its literary merits and relevant to the field of bioethics.

## Opening up *Bloodletting*

Lam's complex rendition of medicine, hospitals and doctors makes the collection appealing for all kinds of readers, from those who cannot tell a crash cart from a cardiogram to health-care insiders who may well recognize their own experiences on the pages of *Bloodletting*. For instance, in "How to Get into Medical School, Part 1", we are introduced to Ming, a no-nonsense and meticulous undergraduate student. She has finished her molecular biology exam characteristically early but is lingering to time her exit from the classroom with Fitzgerald, the artistic study partner with whom she has shared many late nights in the library. Their romantic, post-exam exchange in which Ming gushes to Fitzgerald, "Thank you for explaining the Kreb's cycle to me," will bring a smile of recognition to those for whom undergraduate crushes have developed in the depths of library basements – and for those who have endeavoured to learn the Kreb's cycle.

In "Take All of Murphy", we enter the anatomy cadaver dissection lab, which Lam describes as the "first rite of medical school". We are introduced to Sri, "the sentimental wreck who can't even cut open an arm", and his classmate, Chen. Here, our medical students are confronted with the first of many ethical dilemmas presented throughout the stories. In this case, the students argue over whether or not to cut through the biblical scripture text tattooed on their cadaver. "You should respect a man's symbols", says Chen in response to Ming's insistence that they adhere to the dissection instructions. This is the first of many instances whereby cut-and-dry rules collide with nebulous real-world situations.

By the seventh story, "Eli", our characters are now physicians. Fitzgerald is a trauma doctor juggling a chaotic emergency room when two police officers arrive with their arrested suspect, Eli. He requires medical care for a head wound that is likely the result of police brutality. In this story, Fitzgerald, who was gentle and sincere as a medical student, has transformed into a shrewd and forthright adversary within a system of power struggles. "The game is supposed to go like this", he tells us, by way of introduction to the manoeuvring of various authority figures in his ER. Eli is a physical threat, but it is the police officers whom Fitzgerald approaches with the most cunning and suspicion. Once again, Lam removes the crisp white lab coat from the profession and gives us a view of medicine as marred and, to some extent, unheroic.

By the time we reach "Night Flight" near the end of the collection, Fitzgerald has developed a substance addiction. As a result, he has been forced to leave his position at the hospital and is now employed as a travel physician, a line of work in which he can keep his addiction hidden. However, Fitzgerald's substance use is the least of the ethical concerns raised in this story of a woman attempting to med-evac her dying husband from Guatemala to Canada. Additionally, we are introduced to conflicts between public and private health insurers, rich and poor country medical capacities, and hurtful truth-telling versus compassionate lying.

## *Bloodletting *in cultural and literary context

Throughout these and his other stories, Lam paints a picture of physicians as well-intentioned, partly-competent masters of illusion. They are men and women who hold the pose of confidence and certainty without always possessing it. Their authority, knowledge, and apparent control of the situation are often manufactured to serve professional and, occasionally, personal ends.

For those reasons, the last story's final image functions as a metaphor for the entire collection. After a tiring and frantic night shift in the emergency room, Chen returns home to a darkened bedroom: "The light through the blinds falls diagonally in fat stripes on the floor, and is warm on the carpet whose stains are highlighted and made attractive, important." In Lam's collection, the stains and messes that characterize the practice of medicine are highlighted and made attractive, important. It is the doctors' foul-ups, the valiant attempts, the guesswork, and the emotional turmoil that are made important here, not their enlightenment, their objectivity, nor their cold, clinical perspective.

This version of medical science and its practitioners is crucial and sets it apart from other contemporary renditions that celebrate doctors as infallible knights in shining white-lab-coat armour and science as completely reliable and failsafe. In short, *Bloodletting *is the anti-*CSI*. Whereas the TV-series *CSI *(*Crime Scene Investigation*) presents science as unambiguous, unquestionable and objective, *Bloodletting *reminds us repeatedly that medicine is an inexact science full of guesses that go wrong, messes that are beyond cleaning and problems that cannot be solved with a quick stitch or pill.

Lam foreshadows this idea with the quotation that introduces his collection – "Medicine is a science of uncertainty and an art of probability" – and reinforces it with a glossary of over one hundred medical phrases at the end of the book. Readers unfamiliar with these technical terms may find that their regular reference to this glossary through his early stories fades away as such details become unimportant to the actual challenges being faced by Ming, Fitzgerald, Sri and Chen. The more that we view these medical tools and diagnostics as irrelevant, the more we encounter physicians who are illusionists drawing on a very different set of talents with which to manage scenarios. The glossary thus becomes ironic, signifying not the possibility of full and complete knowledge, but rather its absence and elusiveness.

Those are heady and important ideas and, to Lam's credit, he weaves them into immensely entertaining and compelling stories that have earned him the high praise that he deserves. In the context of popular versions of medicine or science, therefore, the collection is new and exciting. But in the context of Canadian literature, these ideas – the difficulty of objectivity, the trouble with authority, the challenge of so-called clear communication – are familiar ones, already examined with significant complexity by a long line of well-recognized Canadian authors: Mavis Gallant (in her From the Fifteenth District stories), Alice Munro (in Lives of Girls and Women and beyond), and Margaret Atwood (in her Bluebeard's Egg and Wilderness Tips collections). Because these more accomplished authors have already covered similar ideological territory, on occasion Lam's stories appear to be lighter and simpler (but more accessible and more entertaining) versions of their predecessors. "Code Clock," for instance, is a great story, but its conclusion is too quick, too cute, and (almost) too didactic. "An Insistent Tide" suffers a similar fate. But that occasional simplicity alone is not enough to disapprove of Lam's collection; the fact that the comparison to literary heavyweights like Gallant, Munro, and Atwood can be made at all is a tribute to Lam's accomplishment.

## *Bloodletting *as a tool for bioethics training

Along with its literary contribution, *Bloodletting *also has relevance for academic training. Lam's presentation of health care as messy and subjective parallels the messiness of bioethics. While there are clear-cut right and wrong answers to some questions, much of bioethics involves the search for solutions that are most right or least wrong, positions that can change with a shifting point of view. To this end, Lam's stories might find themselves a home in bioethics curricula as training tools. The initial step in ethical decision making is recognition and articulation of issues as moral dilemmas in the first place. Each of his short stories is embedded with ethical quandaries that could act as a springboard for such bioethics teaching.

In "Code Clock", for instance, we see our medical resident responding to a "code blue" for a hospital in-patient with a cardiac arrest. This is a tense and compelling story during which we have access to the resident's inner monologue. His failing confidence and capacity will prompt many readers to consider avoiding teaching hospitals in the future. However, Lam has hit on a real and central challenge in medical training: how to balance the inevitable need to learn new skills with the dire risks associated with incompetence. This story points a spotlight on the medical school mantra of "watch one, do one, teach one", prompting critical reflection on the process of becoming a proficient practitioner. The story is also imbued with counterproductive power dynamics amongst the various physicians and nurses at the scene, providing yet another starting point for ethics discussion with health care trainees.

In "Winston", Lam presents Sri as a medical trainee providing a psychiatric consultation for a young man who believes he has been poisoned. Readers join Sri in his journey from clarity of purpose at the start of the encounter to self-doubt about what is real and what is right by the end of the exchange. Lam gives bioethicists much fodder for discussion. First, he prompts us to consider the challenges associated with judging patients' truthfulness in the face of possible psychosis and, moreover, the consequences of being wrong. Second, in this story and others, he encourages us to question what constitutes appropriate supervision of medical trainees from their overseeing physicians who may be distracted, disinterested or themselves ill-informed. Most important, however, is the issue of boundaries in medicine and the lengths to which health care providers can and should go to provide appropriate care.

Sri's unease with the situation prompts him to step outside of typical behaviour and visit Winston's home where his understanding of the story becomes even more confused. Sri's actions, born from care and concern for Winston's welfare, ultimately lead to a situation of violence and harm for his patient. This story lays bare the conflict of dual loyalty faced by health care providers; that is, commitment to their patients on one hand, while simultaneously acting as society's gatekeepers in line with legislation (in many environments) that authorities be alerted if a person presents a danger to him/herself or others.

"Contact Tracing", a topic of perennial interest to bioethicists, is the title of one further story that provides rich material for ethics training. Here, Lam draws on his experience as a Toronto-based physician during the SARS epidemic in 2003. Supported by World Health Organization press releases and fictitious medical chart entries, Lam presents the experiences of a range of physicians and nurses as they struggle through this devastating experience. His characters suffer the stigmatization of quarantine, the dehumanization of isolation and the sequelae of SARS itself. He describes the impact of one hospital's strategy for selecting and then compelling nurses to serve on their new SARS unit, prompting contemplation amongst readers on health care providers' duty to care and just reciprocity for such service. Like much of this book, "Contact Tracing" is accessible to all but will have a unique impact on those who can associate with the experience – in this instance, a health care response during an infectious outbreak.

## Conclusion

"Dignity and decorum are crucial", says the anatomy instructor in "Take All of Murphy", but they can be difficult to achieve in Lam's world of health care. Accuracy, objectivity and precision are also crucial, but in this collection they, too, are elusive.

*Bloodletting and Miraculous Cures *offers a refreshing, entertaining and sometimes disturbing view of doctors and doctoring that is satisfying to both readers unacquainted with the challenges of medicine and those all too aware of the messy reality of the practice. It is a credit to Lam who uses his imagination and, no doubt, his experience as an emergency room physician to cut beneath the surface and present a collection of stories that brings to light the everyday quandaries faced by health care providers.

